# Enhancing mineral bioavailability from cereals: Current strategies and future perspectives

**DOI:** 10.1111/nbu.12324

**Published:** 2018-05-08

**Authors:** M. F. Aslam, P. R. Ellis, S. E. Berry, G. O. Latunde‐Dada, P. A. Sharp

**Affiliations:** ^1^ King's College London London UK

**Keywords:** aleurone, bioaccessibility, bioavailability, iron, wheat, zinc

## Abstract

Inadequate intake of essential minerals such as iron and zinc is a public health concern in the UK, particularly for girls and young women. Approximately 30% and 50% of the zinc and iron, respectively, in the UK diet is provided by cereals. In wheat, most of the iron and zinc is contained within the aleurone cell layer; however, aleurone is removed during processing of wheat into white flour. While elemental iron powder is added back into white flour at the milling stage, there is no restoration of zinc. Elemental iron powder has very low bioavailability, and therefore, in our current Biotechnology and Biological Sciences Research Council Diet and Health Research Industry Club‐funded project, we are investigating the potential use of aleurone as a bioavailable source of minerals that could be added to wheat‐based foods. This work has relevance for the food industry and may establish the use of aleurone as a functional food ingredient for fortification of a range of cereal‐based food products.

## Cereals as an important source of minerals

Iron deficiency and zinc deficiency are global nutritional problems. It is estimated that up to 33% of the world population are iron deficient (Zimmermann & Hurrell 2007) and 25% are at risk of chronic zinc deficiency (Maret & Sandstead 2006). In the UK, adolescents and adult females have a higher risk of low status in these metals due to low dietary intake (Bates *et al*. 2016). Given that iron and zinc are found in similar foods and the bioavailability of both metals is regulated by common dietary factors, it is likely that diets that are either deficient or have low bioavailability of one of these metals will also be a poor nutritional source of the other metal. Hence, low iron and zinc status may co‐exist in certain UK population groups; indeed, there is good evidence from studies in the US that low serum ferritin is associated with smaller zinc pools in pre‐menopausal women (Yokoi *et al*. 2007), and that serum zinc positively correlates with haemoglobin levels in other population groups (Brown *et al*. 2004).

Cereals and cereal products are important dietary sources of several minerals. Data from the *National Diet and Nutrition Survey (NDNS)* Rolling Programme (years 5–6) (Bates *et al*. 2016) indicate that cereals provide an average of 25% of the zinc, 31% of the calcium and 39% of the iron in the UK diet. For those consuming a largely plant‐based diet, intakes of minerals from cereals are substantially greater than from an omnivorous diet (56% of zinc, 49% of calcium and 28% of iron in diet are provided by a combination of meat, fish and dairy). The contribution of cereals to mineral intakes in certain age groups is of major importance; for example, in children and adolescents (aged 1.5–18 years) cereals contribute more than 50% of total dietary iron intake (Table 1). Wheat is the most commonly consumed cereal in the UK. The National Association of British and Irish Millers estimates that approximately 5 million tonnes of wheat are milled annually producing 4 million tonnes of flour. The majority of this flour (60%) is used to make bread, and it is estimated that the average annual intake of wheat flour in 2016–2017 was 59 kg/person.

**Table 1:** The percentage contribution of cereals to mineral intakes in different age groups in the UK. Data are taken from the National Diet and Nutrition Survey Rolling Programme, years 5–6 (Bates *et al*. 2016)

Mineral	Age group (years)				
	1.5–3	4–10	11–18	19–64	65+
	%	%	%	%	%
Calcium	22	34	38	31	30
Iron	51	54	51	40	41
Zinc	25	29	30	26	24

**Figure 1:**
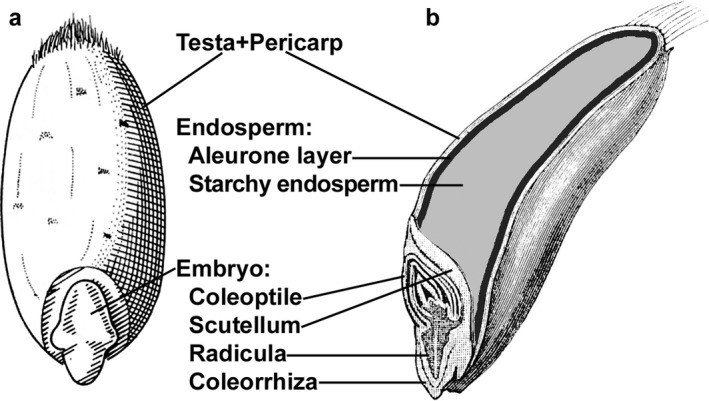
Structure of the wheat (*Triticum aestivum*) caryopsis. (a) The structure of the mature wheat grain. (b) The position of the aleurone layer relative to the testa and pericarp, and the starchy endosperm. Graphics are taken from The Seed Biology Place (http://seedbiology.de) and are reproduced with the kind permission of Professor Gerhard Leubner, Royal Holloway, University of London.

The wheat grain comprises a number of highly specialised structures (Fig. 1). During milling, the outer ‘bran’ is separated from the endosperm, which is rich in starch and protein and is used to produce white flour. The bran is formed of a number of layers including the testa and pericarp and aleurone layers. While aleurone is considered to be part of the endosperm from a botanical perspective, it is strongly attached to the nucellar epidermis and is removed along with the bran and embryo during milling. Bran is rich in both vitamins and minerals, with the majority of these nutrients residing in the aleurone component (Neal *et al*. 2013; Wu *et al*. 2013; Latunde‐Dada *et al*. 2014; De Brier *et al*. 2016). Iron and zinc in particular are abundant in aleurone purified from wholewheat flour (Latunde‐Dada *et al*. 2014).

Iron and zinc may be present in wheat in a number of different forms. Studies have found that a significant proportion of wheat iron and zinc is bound to nicotianamine (Eagling *et al*. 2014); however, it is unclear whether this form exists in the aleurone layer. Using X‐ray fluorescence, iron and zinc have been co‐localised with phosphorus in the aleurone layer (Neal *et al*. 2013; De Brier *et al*. 2016), and higher resolution secondary ion mass spectrometry has demonstrated that iron is specifically located in phytin globoids in aleurone cells (Moore *et al*. 2012). These findings indicate that iron and zinc exist predominantly as phytate complexes within the aleurone cells. Phytate is a major inhibitor of iron and zinc absorption, and therefore, bioavailability of these minerals from wheat‐based foods is low (Hallberg *et al*. 1989; Gibson *et al*. 2010).

## Current fortification strategies

In the 1950s, Bread and Flour Regulations were introduced into legislation to enforce the mandatory fortification of white and brown flour with calcium, iron, niacin and thiamine, which are lost during milling. Iron is added to white and brown flours at the mill at prescribed levels (1.65 mg/100 g flour) to restore iron to levels present in an 80% extraction of wholegrain flour. Despite sub‐optimal zinc intake in some UK population groups, there is no current recommendation to add zinc to white flour.

The benefits of country‐wide iron fortification programmes are unclear. For example, Denmark, which ceased adding iron to cereal flour in 1987, has seen no increase in the incidence of iron deficiency or anaemia in the intervening years (Milman *et al*. 2000). Data on the efficacy of food fortification programmes in other countries were reviewed in the 2010 Scientific Advisory Committee on Nutrition (SACN) report on *Iron and Health* and it was concluded that there was insufficient evidence to demonstrate a definite health benefit (SACN 2010). While there is evidence from developing countries, where iron deficiency anaemia is highly prevalent, that fortification of foods with iron may be an effective strategy to combat deficiency, there are no data available to suggest that iron added to flour in the UK is sufficiently bioavailable to be of benefit for groups at risk of developing iron deficiency or anaemia. However, a position statement from SACN supported the continued fortification of wheat flour with iron (SACN 2012) on the basis that its removal would decrease overall iron intake in the general population and increase the number of individuals in specific population groups whose intakes would fall below the lower reference nutrient intake (LRNI). The latest *NDNS* data indicate that 48% of girls aged 11–18 years and 27% of adult females aged 19–64 years have iron intakes below the LRNI (Bates *et al*. 2016). SACN therefore recommended that there is still a strong case for retaining the mandatory addition of iron to wheat flour.

A range of iron compounds have been approved by the European Union for food fortification, and it is assumed that the absorption and utilisation of iron fortificants is similar to endogenous food iron. However, these compounds differ in both solubility (Hurrell 1997) and relative bioavailability (Hurrell 2002). In the UK, elemental iron powder is the main form of iron used to restore white flour or to fortify other cereal products; however, elemental iron has poor solubility in the gastrointestinal tract and therefore has low bioavailability (Hurrell 2002). Fairweather‐Tait and colleagues compared the bioavailability of hydrogen‐reduced elemental iron powder with other iron compounds from a fortified breakfast cereal meal and found that the inclusion of sodium‐iron‐ethylenediaminetetraacetic acid (Na_2_FeEDTA), a commonly used iron fortificant, significantly increased iron bioavailability compared with hydrogen‐reduced elemental iron powder alone (Fairweather‐Tait *et al*. 2001). In addition, an *in vitro* study comparing iron availability from fourteen iron sources added to a wheat‐based breakfast cereal found that most compounds increased ferritin formation in Caco‐2 cells (a surrogate marker for iron absorption and storage in Caco‐2 cells), with Na_2_FeEDTA giving the greatest ferritin response (Wortley *et al*. 2005). Interestingly, the addition of milk or coffee to the breakfast cereal meal significantly reduced iron availability. This finding highlights that the form of iron present in food and its interactions with other food components are the main determinants of iron bioavailability.

There is no mandatory requirement to add iron to wholegrain cereal products (which retain the bran, comprising the aleurone, pericarp and testa layers of grain); however, the bioavailability of endogenous iron (and zinc) from these products is unclear. The encapsulation of nutrients by physically intact cell walls in the cells of edible plant tissues (*e.g*. leguminous seeds and cereal grains) has been shown to hinder nutrient bioaccessibility (*i.e*. the release of minerals from the food matrix) (Ellis *et al*. 2004; Grundy *et al*. 2015). Our recent data indicate that plant cell walls in wheat remain largely intact during food processing, mastication and intestinal digestion, suggesting that mineral bioaccessibility from these cells may be limited (Latunde‐Dada *et al*. 2014; Edwards *et al*. 2015). Thus, a combination of physical inaccessibility of minerals encapsulated by plant cell walls, the removal of aleurone during milling, poor bioavailability of currently used fortificants and high levels of inhibitory phytates and phenolic compounds (Hallberg *et al*. 1989; Hurrell *et al*. 2002; Gibson *et al*. 2010; Brnic *et al*. 2014) may serve to limit absorption of iron and zinc from cereal‐based foods. In summary, fortification of foods with iron and zinc remains a major challenge and novel approaches to improve mineral bioaccessibility and bioavailability in cereals are required as part of the solution to low intake of these nutrients both in the UK and worldwide.

## Future perspectives

The nutrient composition of aleurone suggests that this layer may have potential for use as a source of vitamins and minerals to fortify cereal‐based foods. White bread containing 20% aleurone has comparable micronutrient content to wholewheat bread (Brouns *et al*. 2012). Furthermore, human feeding studies have shown that the bioavailability of folate (Fenech *et al*. 1999, 2005), and plasma levels of betaine and other methyl donors (Keaveney *et al*. 2015) are enhanced following consumption of aleurone‐enriched breads. Interestingly, a recent *in vitro* study has suggested that iron availability from wholewheat bread is equal to or even greater than that from fortified white bread (Nikooyeh & Neyestani 2017). To determine whether aleurone may provide a bioavailable source of iron, we compared iron availability *in vitro* (using ferritin formation in Caco‐2 cells as a marker for iron absorption and storage within cells) from purified aleurone and wholewheat flour (provided by Bühler AG, Switzerland) (Latunde‐Dada *et al*. 2014). Iron absorption was greater from the purified aleurone flour, highlighting the potential for this cereal fraction to deliver minerals in a bioavailable form.

Given that plant cell walls are highly resistant to digestion in the gastrointestinal tract, we hypothesised that disruption of wheat aleurone cell walls prior to food manufacturing might further increase mineral bioavailability. Treatment of purified aleurone with Driselase (a research‐grade enzyme preparation containing a combination of xylanase, lamarinase and cellulase) resulted in partial digestion of aleurone and increased uptake of iron by Caco‐2 cells (Latunde‐Dada *et al*. 2014). This raises the prospect that food‐grade enzymes commonly used in baking may also be of benefit in increasing mineral bioavailability from wheat‐based foods. In support of this possibility, the use of xylanases, which breakdown the arabinoxylan component of the aleurone cell walls, can increase iron bioaccessibility from sorghum (Baye *et al*. 2015). Changes in bread‐making technology may also have a role to play. For example, recent work indicates that sourdough breads, which have a longer fermentation time, have greatly reduced phytate content compared with bread made using the Chorleywood Bread Process resulting in increased iron availability (Rodriguez‐Ramiro *et al*. 2017).

Physical disruption of aleurone cell walls may also increase mineral bioaccessibility. Standard milled bread flour has a particle size in the range 100–200 μm and aleurone cells a diameter of 50–75 μm. We therefore studied the effects of micro‐milling (to achieve particle sizes 10–20 μm) on iron availability from wheat flour. Micro‐milling resulted in disruption of the aleurone cell walls, and importantly, there was no loss of mineral content in the micro‐milled flour compared with flour produced by standard milling methods. Iron solubility and availability from the micro‐milled flour was increased in our *in vitro* assay (Latunde‐Dada *et al*. 2014).

While approaches to disrupt the aleurone cell walls either chemically or physically may be effective in increasing mineral bioaccessibility, potentially there may be adverse effects on iron and zinc bioavailability in foods. For example, ball‐milling to decrease flour particle size has been shown to increase the extractability of phytate (an inhibitor of iron and zinc absorption) from the aleurone layer (Antoine *et al*. 2004). Furthermore, the arabinoxylan component of plant cell walls is highly conjugated with phenolic acids (also inhibitors of iron and zinc absorption), including ferulic acid (Brouns *et al*. 2012), and micro‐milling bran increases release of phenolic acids (Hemery *et al*. 2010) from the plant cell walls. It is possible that the use of xylanases in food preparation may have a similar effect. Thus, while disruption of aleurone cell walls may increase mineral bioaccessibility, the concomitant increase in the release of phytate and ferulic acid may counteract any beneficial effects.

In our current Biotechnology and Biological Sciences Research Council (BBSRC) Diet and Health Research Industry Club (DRINC)‐funded project ‘Increasing micronutrient bioaccessibility from wheat’, we are investigating the potential use of aleurone as a bioavailable source of minerals in wheat‐based foods. There is an ongoing debate over the efficacy of current iron fortification strategies. While there is no current requirement to fortify flour with zinc in the UK, strategies to increase zinc bioavailability would benefit a number of population groups. Our approach to fortification is novel and seeks to utilise the endogenous iron and zinc contained within the wheat aleurone rather than through the addition of metal salts or elemental powders to flour. While our proposal focuses on iron and zinc in wheat, the work has potential application to other vitamins and minerals found at high levels in the aleurone layer, and for other cereals. If successful, our data may establish the use of aleurone as a functional food ingredient and could lead to the development of a range of cereals‐based food products with enhanced nutritional quality and potential health benefits for groups with poor iron and zinc status.

## Conflicts of interest

The authors have no conflicts of interest to disclose.
